# Prediction of the COVID-19 outbreak in China based on a new stochastic dynamic model

**DOI:** 10.1038/s41598-020-76630-0

**Published:** 2020-12-09

**Authors:** Yuan Zhang, Chong You, Zhenhao Cai, Jiarui Sun, Wenjie Hu, Xiao-Hua Zhou

**Affiliations:** 1 School of Mathematical Sciences, Peking University, Beijing, 100871 China; 2 Center for Statistical Sciences, Peking University, Beijing, 100871 China; 3 Beijing International Center for Mathematical Research, Peking University, Beijing, 100871 China; 4 Department of Biostatistics, School of Public Health Peking University, Beijing, 100871 China

**Keywords:** Infectious diseases, Applied mathematics

## Abstract

The current outbreak of coronavirus disease 2019 (COVID-19) has become a global crisis due to its quick and wide spread over the world. A good understanding of the dynamic of the disease would greatly enhance the control and prevention of COVID19. However, to the best of our knowledge, the unique features of the outbreak have limited the applications of all existing dynamic models. In this paper, a novel stochastic model was proposed aiming to account for the unique transmission dynamics of COVID-19 and capture the effects of intervention measures implemented in Mainland China. We found that: (1) instead of aberration, there was a remarkable amount of asymptomatic virus carriers, (2) a virus carrier with symptoms was approximately twice more likely to pass the disease to others than that of an asymptomatic virus carrier, (3) the transmission rate reduced significantly since the implementation of control measures in Mainland China, and (4) it was expected that the epidemic outbreak would be contained by early March in the selected provinces and cities in China.

## Introduction

The current outbreak of coronavirus disease 2019 (COVID-19) has become a global crisis due to its quick and wide spread over the world. As of August 24, 2020, the outbreak of COVID-19 has caused 84,981 confirmed cases and 4634 fatalities in Mainland China^1^. For the purpose of control and prevention, various containment measures have been implemented in Mainland China since January 19, 2020, including traffic restrictions, contact tracing, mandatory face masks in public spaces, entry or exit screening, isolation, quarantine and awareness campaigns. Especially on January 23, 2020, a strict travel restriction was introduced in Wuhan, Hubei province, and the city has been locked down since then^2^.

A good understanding of the epidemic dynamic would greatly enhance the control and prevention of COVID-19 as well as other infectious diseases, while dynamic model is probably one of the oldest mathematical tools to study the law of epidemic development whose history can be traced backed to the well-known Susceptible–Infected–Removed (SIR) model proposed in 1950s^3^. Due to the usefulness and advantage in prediction, and especially inference, SIR and its modified models are still widely applied in the study of SARS^4^, H1N1^5^, and particularly the COVID-19 pandemic^6–9^. We hereby present a brief review on some of the representative works as follows.

Recently, Tang et al.^10^ proposed a deterministic compartmental model by taking the clinical progression, epidemiological status, and the intervention measures into account. However, it implicitly assumed that the disease is not infectious during incubation period, which is not the case in COVID-19. In addition, it assumed that quarantine was implemented as soon as the infection occurred, which fails to reflect the inevitable latency brought by medical tracking. In the study of Wu et al.^11^, it proposed an extended SEIR model by considering transmissions among cities. However, it did not take the control measures into account such as tracing and quarantine. Furthermore, it also assumed that COVID-19 is not infectious before symptoms onset. For more similar or simple deterministic ODE models to COVID-19, we refer to Liu et al.^12^ for an overview. Yang et al.^13^ employed a discrete time difference equation (DE) model to predict the epidemic trend of COVID-19. The proposed model correctly took the infectious incubation into account. However, this model did not consider the time needed for medical tracking or the time lag between symptoms onset and diagnosis. Besides, the rationale behind the assumption of the equal transmission probability between symptomatic and asymptomatic virus carriers was questionable.

In contrast to the deterministic models (ODE or DE) summarized above, the transmission of disease between individuals in real world is inevitably random in nature. As a result, numerous stochastic dynamics models have been developed since the pioneering randomization of SIR model^14^. In fact, a deterministic ODE model can often be seen as the mean-field equation of the corresponding stochastic counterpart. Under certain conditions, the mean-field equation may represent the evolution of the expectation of the corresponding stochastic model. In some more generalized cases, the mean-field equation is a large scale approximation of the corresponding stochastic model, which can be seen as a process version of *Law* *of* *Large* *Numbers*. However, if the size of outbreak is not comparable to that of the total population, the randomness is more significant, and hence a stochastic model is a better choice to quantify the uncertainty in estimates and predictions in such case. Furthermore, stochastic dynamic model is also known for its expandability to incorporate individual variations^15^, or even spatial structures^16^, which may not be fully captured by its mean-field equations. To our knowledge, stochastic dynamic modeling for COVID-19 is yet relatively rare comparing to its deterministic counterparts, though preliminary approaches such as statistic exponential growth models were considered in recent studies^17,18^. Recently in the study of Chinazzi et al.^19^, an existing discrete time stochastic model was employed to estimate the “effect of travel restrictions on the spread” of COVID-19. However, the unique features of COVID-19, such as the infectious incubation and asymptomatic carriers, as well as control measures such as medical tracking, are still yet to be captured in their work.

To remedy the aforementioned issues in the existing studies, and depict a more realistic transmission mechanism, we propose a novel stochastic compartmental model which captures the unique transmission dynamics of COVID-19 and the effects of intervention measures implemented in Mainland China. Our proposed stochastic model aims to study the COVID-19 outbreak in the following aspects: (1) estimation of key epidemiology parameters; (2) prediction of epidemic development; (3) estimation of unobservable carriers and epidemic containment date; and (4) assessment of control measures.

The rest of this paper is structured as follows. In “Methods” section we describe the data used in this study, and introduce the proposed stochastic dynamic model and parameter estimations. “Results” section presents our findings. We discuss our results, advantages and limitations in “Discussion” section.

## Methods

### Data sources

Data used in this study include numbers of confirmed diagnosis, recoveries and fatalities in the following major provinces and cities of China: Beijing, Shanghai, Chongqing, Guangdong, Zhejiang and Hunan. These public available data were retrieved from local Health Commission based on a daily update^20–25^. The corresponding population of residents in each region is collected from China National Bureau of Statistics^26^. Note that we exclude Hubei province which was the epicenter due to the following reasons: (1) the medical resources in Hubei province were overburdened at the beginning of the epidemic, and not all individual with confirmed diagnosis could get immediate hospitalization; (2) the diagnostic criteria were changed overtime in Hubei which resulted in a massive surge of confirmed cases in mid February^27^; and (3) the fatality rate in Hubei province was much higher than other regions in China. These features distinct the dynamic model in Hubei from other regions of China, which should be considered in our future studies.

### Model description

In our study, none of selected provinces and cities has more than 2000 accumulated confirmed cases by now (see table S3 in Supplementary F). These number, though alerting, are not comparable to the total population in provinces or cities, which are of an order 10 million100 million (see Table S4 in Supplementary F). Hence, a novel stochastic dynamic model is designed to capture the unique features of the COVID-19 outbreak, where the unique features here refer to

1. Infectious incubation period: unlike SARS, COVID-19 is infectious before symptoms onset^28^.
2. Large portion of asymptomatic virus carriers: it has been found that the proportion of asymptomatic infected population is non-negligible^29^.
3. Unprecedented contact control and medical tracking measures: various containment measures have been implemented in Mainland China since January 19, 2020; especially on January 23, strict travel restriction was introduced in Wuhan at an unprecedented scale, and the city has been locked down since then^2^; at the same time, great efforts have also been taken contact tracing and quarantine, for example, forty thousand close contacts was successfully tracked more than in Zhejiang Province^30,31^.

To our knowledge, these features have not yet been fully captured by the existing stochastic dynamic models for the epidemic.

Under mild assumptions that (1) motions of all individuals in the system are independent, and (2) the total population in the system is a fixed number of *N*, we propose a new stochastic model with state variables *S*,* E*,* Q*,* IN*,* IH*,* R* and *D* which stand for susceptible, exposed, quarantined, symptomatic, hospitalized, recovered and dead population respectively. Some states can be further divided into substates, see Supplementary A for more detail. Note that each individual can be classified into one of the above states at a specific time. The evolution of population size in each state over time forms a continuous time Markov Process can be described as follows:

i. Infection: Every infected case in *E* or *IN* passes a pathogen to its secondary case at Poisson rate *λ*_*E*_ = *λ*_*IN*_*θ* or *λ*_*IN*_ respectively. To be specific, a primary case chooses an individual randomly from the total population, and the individual would be infected if it is of state *S*. At each transmission event,
  with probability *ρ*, the secondary case would be symptomatic in the future at a Poisson rate of *r*_*s*_, meanwhile, this contact is traceable with probability *q*;
  with probability 1 − *ρ*, the secondary case would NOT be symptomatic in the future meanwhile, this contact is traceable with probability *q*.
ii. Quarantine: If the contact is traceable, the corresponding secondary case would be quarantined, namely lose its infectivity, at a Poisson rate of *r*_*q*_. Note we assume such individuals would be quarantined or hospitalized till recovery or death.
iii. Hospitalization: Every symptomatic patient in *IN* would be admitted to hospital (IH) at a Poisson rate of *r*_*H*_. With probability *p*_*l*_, it would be a light/mild case and with probability 1 − *p*_*l*_, it would be a severe case.
iv. Symptoms relief: A severe case relieves symptoms to light/mild symptoms at a Poisson rate of *r*_*b*_.
v. Recovery: Asymptomatic patients, symptomatic but yet hospitalized patients and hospitalized patient with light/mild symptoms would recover at Poisson rates of *γ*_*A*_, *γ*_*IN*_ and *γ*_*IH*_ respectively.
vi. Death: Symptomatic but yet hospitalized patients and hospitalized patient with serve symptoms would die at Poisson rates of *δ*_*IN*_ and *δ*_*IH*_ respectively.

The process can be illustrated by Figure S1 in Supplementary A.

### Estimation of model parameters

**Figure 1:**
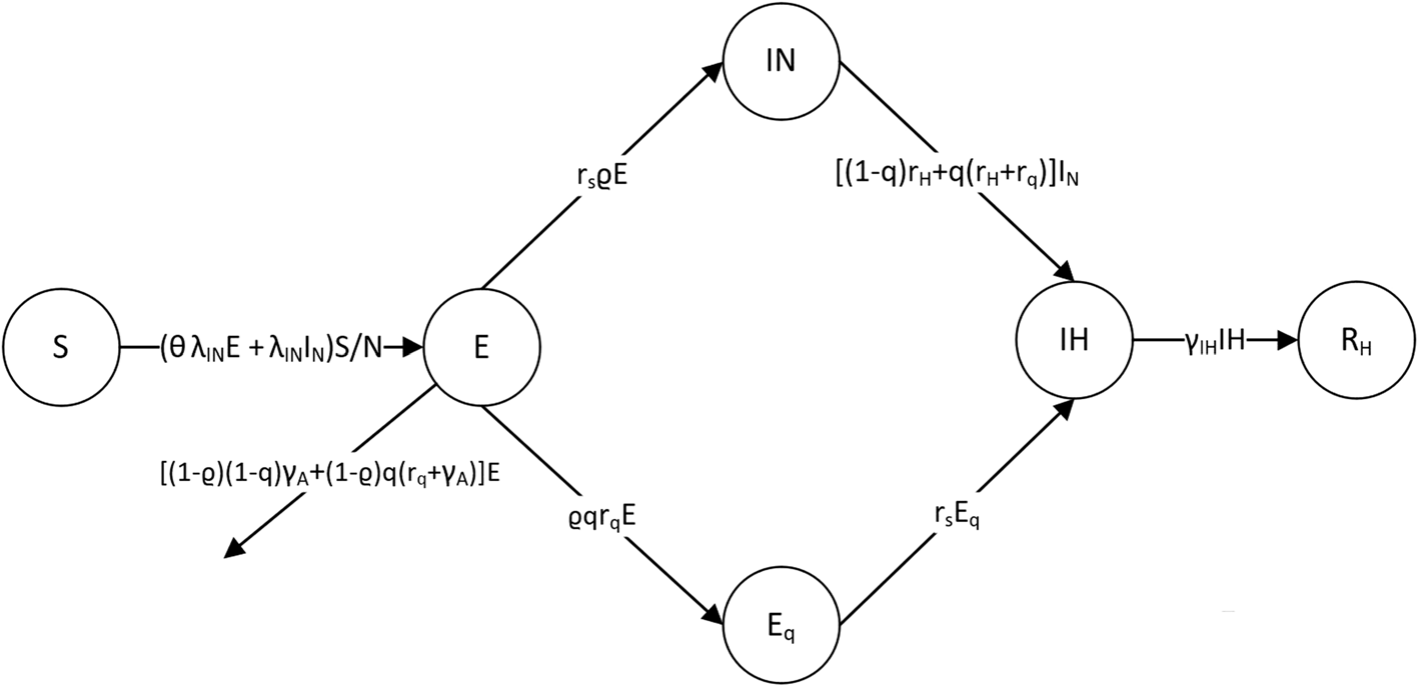
States-collapsed version of the stochastic process.

The proposed model in “Model description” section provides a comprehensive and realistic description to the transmission mechanism of the current outbreak of COVID-19. However, with limited information retrieved from the public available data, a state-collapsed version of the stochastic process in Fig. 1 is used for the purpose of parameter estimation, which would ease the identifications of the initial values in the model. See Supplementary B for rationale behind such simplification.

The sizes of *IH* and a substate of *R* (namely *R*_*H*_ in Supplementary A) over time *t* can be observed directly from the collected data, that is, the number of existing confirmed cases and reported recoveries at *t* respectively; while the remaining states are latent, namely not observable. Among the latent states, the initial value *S*(0) can be approximated by the population of permanent residents in the city or province, *E*_*q*_(0) is zero as there was no quarantine implemented before January 23, 2020 and *R*_*N*_(0) can be set to any number as it would not affect the estimation and prediction of the model. However, the initial values, *IN*(0) and *E*(0), are also non-observable and could be a challenge to determine a priori^32^. In this study, *IN*(0) and *E*(0) are treated as unknown parameters and to be estimated together with other model parameters as described below.

There is a total of 9 model parameters in the proposed model for each selected region. They are *λ*_*IN*_, *θ*_*E*_, *ρ*, *q*, *γ*_*IH*_, *γ*_*A*_, *r*_*s*_, *r*_*q*_ and *r*_*H*_, among which, *r*_*s*_, *r*_*q*_ and *r*_*H*_ are related to the clinical characteristics of the disease and can be prefixed through existing studies. To be more specific, *r*_*H*_ is the inverse of the average time from symptoms onset to diagnosis, *r*_*s*_ is the inverse of the mean incubation period, while *r*_*q*_ is the inverse of mean difference between infectious period and serial interval. Based on preliminary trials, we find that there is very limited information of *γ*_*A*_ which can be obtained from the data, and the estimate is highly influenced by the choices of prior. A possible explanation is that *γ*_*A*_ is less related to the observations. Hence, instead of estimating *γ*_*A*_ with large uncertainty, we prefix *γ*_*A*_ = 1*/*10. Sensitivity analysis is conducted on the different choices of *γ*_*A*_.

The rest of parameters would be estimated from the model. The parameters, *ρ* and *θ*_*E*_, are directly related to the nature of the disease, and hence are considered as constants in China, while, *λ*_*IN*_, *q* and *γ*_*IH*_ may vary in different regions depending the local medical resources, population densities and containment measures. Furthermore, it is more realistic to consider *λ*_*IN*_ and *γ*_*IH*_ as time varying parameters to reflect the effect of intervention measures and improvement of the medical treatment. In this study, a simple setting for the time varying function is used, that is, *λ*_*IN*_(*t*) = 1_{*t*<*T*1}_*λ*_*IN*_ + 1_{*t*>*T*1}_*aλ*_*IN*_ and *γ*_*IH*_(*t*) = 1_{*t*<*T*2}_*bγ*_*IH*_ + 1_{*t*>*T*2}_*γ*_*IH*_. The time *T*_1_ is set to be January 29 as there was an obvious change of rates occurred on January 29 illustrated in Figure 2 of You et al.^33^ We use the observed $\frac{{\Delta R_{{H_{t} }} }}{IH(t)}$ to approximate *γ*_*IH*_ on day *t*, where ∆*R*_*Ht*_ = *R*_*H*_(*t* + 1) − *R*_*H*_(*t*). The time *T*_2_ is selected to be the time when *γ*_*IH*_ has a significant change for each province or city. See Supplementary C for more detailed estimation method including the construction of likelihood functions.

## Results

### Parameter estimations

A summary of the estimated model parameters is given in Table 1, from which we find that

1. The estimate of *ρ* is not sensitive to the choice of *γ*_*A*_, about 30% infected individuals are asymptomatic.
2. The estimate of *θ* decreases as *γ*_*A*_ decreases, but the change is not significant, patients with symptoms are about twice as likely to pass a pathogen to others as asymptomatic virus carriers.
3. The estimate of *q* increases slightly as *γ*_*A*_ decreases. Zhejiang has the highest *q* in the selected regions, which is consistent with the remarkable efforts made by the Government of Zhejiang, which till March 2, 2020 has successfully tracked more than 40,000 close contacts^30^.
4. The estimated initial populations for states *E* and *IN* in each region vary over different choices of *γ*_*A*_, but are still within the same order of magnitude.
5. The estimates of *λ*_*IN*_ and *a* change slightly over different choice of *γ*_*A*_.

**Table 1:** Parameter estimation.

	*γ*_*A*=1/7_	*γ*_*A*=1/10_	*γ*_*A*=1/14_		*γ*_*A*=1/7_	*γ*_*A*=1/10_	*γ*_*A*=1/14_
ρ	0.7259	0.6755	0.6985	θ	0.5189	0.4675	0.4379
λ_IN_^gd^	0.5376	0.5604	0.5712	λ_IN_^zj^	0.4458	0.4926	0.4837
λ_IN_^hn^	0.4589	0.4898	0.4905	λ_IN_^bj^	0.4084	0.4161	0.4334
λ_IN_^sh^	0.4526	0.4606	0.4455	λ_IN_^cq^	0.3394	0.3469	0.3538
q^gd^	0.3475	0.3881	0.4093	q^zj^	0.6727	0.7367	0.7361
q^hn^	0.2960	0.3359	0.3533	q^bj^	0.2459	0.2530	0.2810
q^sh^	0.4946	0.4888	0.4844	q^cq^	0.1840	0.2050	0.2355
a^gd^	0.0508	0.0540	0.0551	a^zj^	0.1277	0.1255	0.1234
a^hn^	0.0245	0.0244	0.0245	a^bj^	0.1066	0.0986	0.0977
a^sh^	0.0967	0.0836	0.0799	a^cq^	0.1162	0.1111	0.1180
E_0_^gd^	190	220	204	E_0_^zj^	334	339	343
E_0_^hn^	254	261	258	E_0_^bj^	60	72	60
E_0_^sh^	73	82	86	E_0_^cq^	111	126	114
IN_0_^gd^	83	77	78	IN_0_^zj^	67	68	64
IN_0_^hn^	40	40	38	IN_0_^bj^	50	48	50
IN_0_^sh^	22	22	20	IN_0_^cq^	87	86	86
γ_IH_^gd^	0.0511	0.0511	0.0511	γ_IH_^zj^	0.0535	0.0535	0.0535
γ_IH_^hn^	0.0787	0.0787	0.0787	γ_IH_^bj^	0.0367	0.0367	0.0367
γ_IH_^sh^	0.1174	0.1174	0.1174	γ_IH_^cq^	0.0709	0.0709	0.0709
b^gd^	0.1542	0.1542	0.1542	b^zj^	0.2625	0.2625	0.2625
b^hn^	0.3995	0.3995	0.3995	b^bj^	0.4433	0.4433	0.4433
b^sh^	0.1492	0.1492	0.1492	b^cq^	0.2193	0.2193	0.2193

### Prediction of the epidemic trend

Based on the estimated parameters, trajectories of the epidemic are simulated using the proposed stochastic dynamic model. For each region, 1000 simulations are conducted to produce the 95% confidence interval for the epidemic evolution of some key populations. In this section, the predictions of populations of states, the containment time of the outbreak, the controlled reproduction number *R*_*c*_, and a test on the effectiveness of the current medical tracking policy are reported.

**Figure 2:**
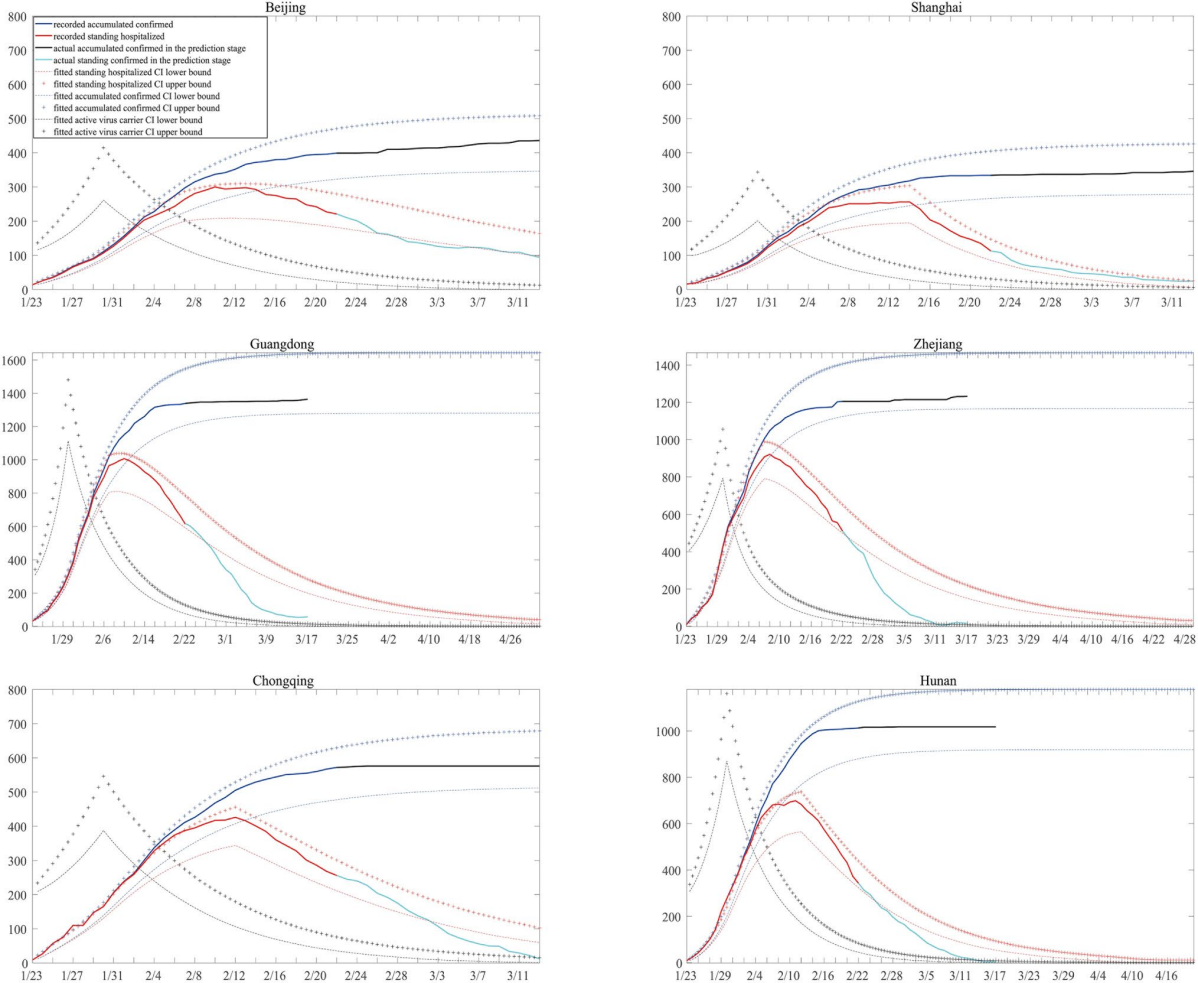
Predicted confidence interval for key states.

Figure 2 plots the 95% confidence interval of:

1. accumulated confirmed cases, namely, the sum of *IH,R*_*H*_ and *D*.
2. the population of state *IH*, representing the people in hospitals.
3. population of active virus carriers, consisting of the states *E* and *IN*.

Note that the first two populations can be directly observed, while populations of *E* and *IN* is not observable. Note that despite more data are now available since the first submission of this work, we decide to use data collected before February 22 for model fitting. This is because that the first wave of COVID-19 pandemic in China has been under good control since mid February, and the number of daily confirmed cases was under 5 in most provinces after February 22. The collected data after February 22 would be used for evaluation of the fitted model. In Fig. 2, the observed accumulated numbers of confirmed cases perfectly lie in the calculated confidence intervals of, while the number of standing hospitalized cases seems to be overestimated. A possible explanation to it is that the mean recover time was shortened at later stage due to the improvement in treatment. Nonetheless, our model provides a good understanding towards the transmission mechanism of COVID-19 in China.

**Figure 3:**
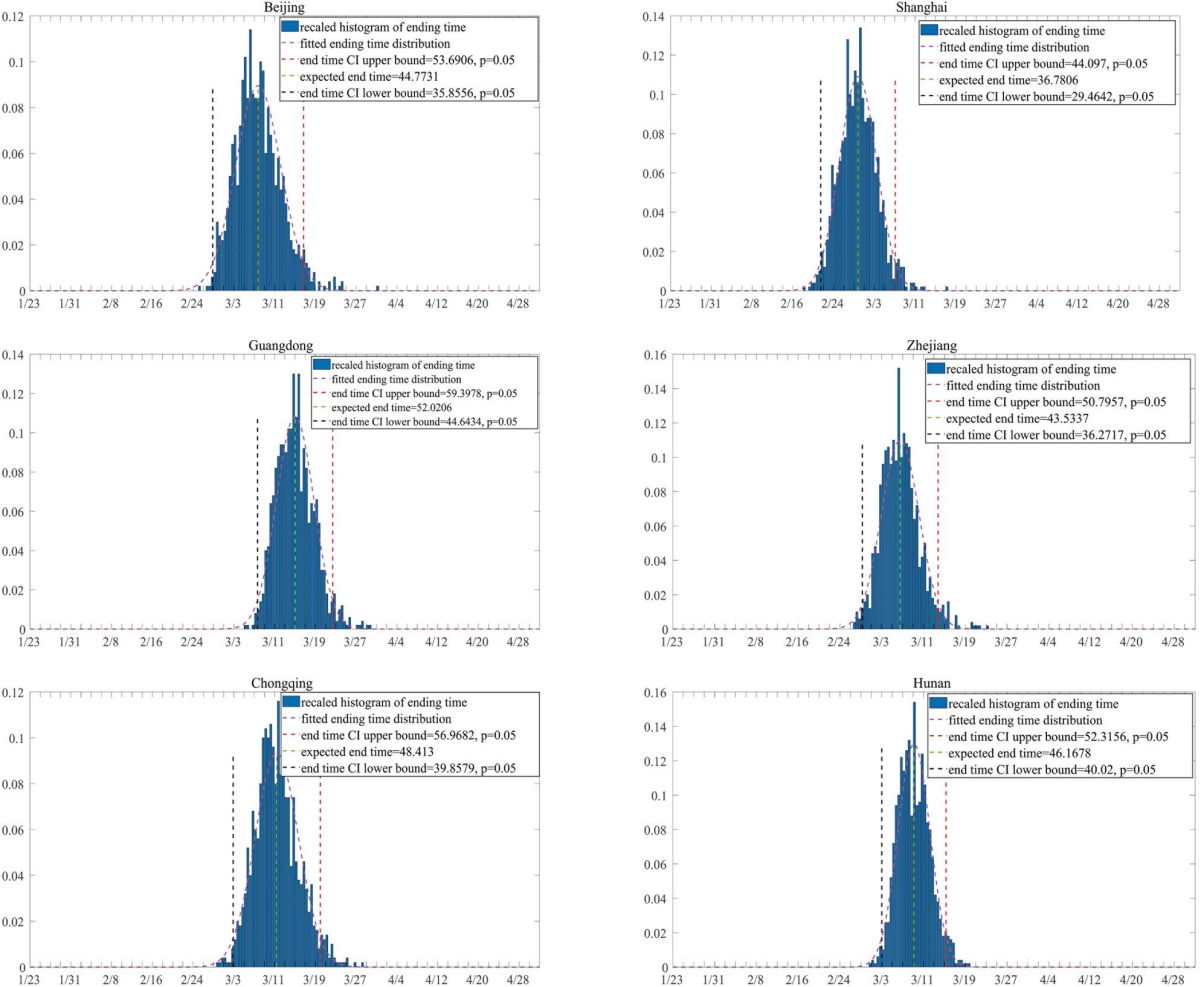
Prediction of the containment time of the outbreak. The containment time of 1000 simulations is plotted as a histogram and is fitted with normal distribution for each region. The *y* axis represents the density of containment time.

The containment time of the outbreak is defined as the time when the number of active virus carriers is, for the first time, less than a threshold *T*_*c*_, here we let *T*_*c*_ = 10. Figure 3 shows the 95% confidence interval of the containment time of the outbreak for each region. Among the six regions in this study, Shanghai is predicted to have the earliest containment time of February 28, while the containment time in Guangdong is predicted to be the latest, around March 15. Comparing the prediction with the observed data, we find that in Beijing, Shanghai and Guangdong, the prediction is consistent while in Chongqing, Hunan and Zhejiang, the containment time is slightly overestimated.

**Figure 4:**
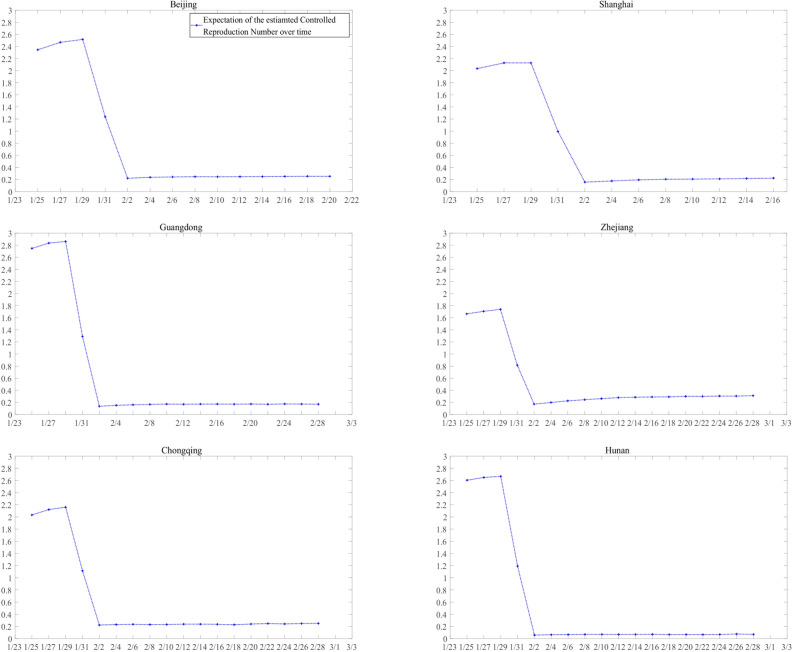
Predicted time-varying *R*_*c*_ curve.

The controlled reproduction number, *R*_*c*_, reflecting the transmission ability of the epidemic, is one of the most important quantities in epidemiology. We refer readers to Supplementary D for the approximation of *R*_*c*_ in this study. Simulation results for the approximated *R*_*c*_ in each region is in Fig. 4. In most provinces and cities our estimated *R*_*c*_ is between 2 and 3 before control measures, and it drops rapidly to about 0.2 between January 29 and February 1 in all selected regions.

**Figure 5:**
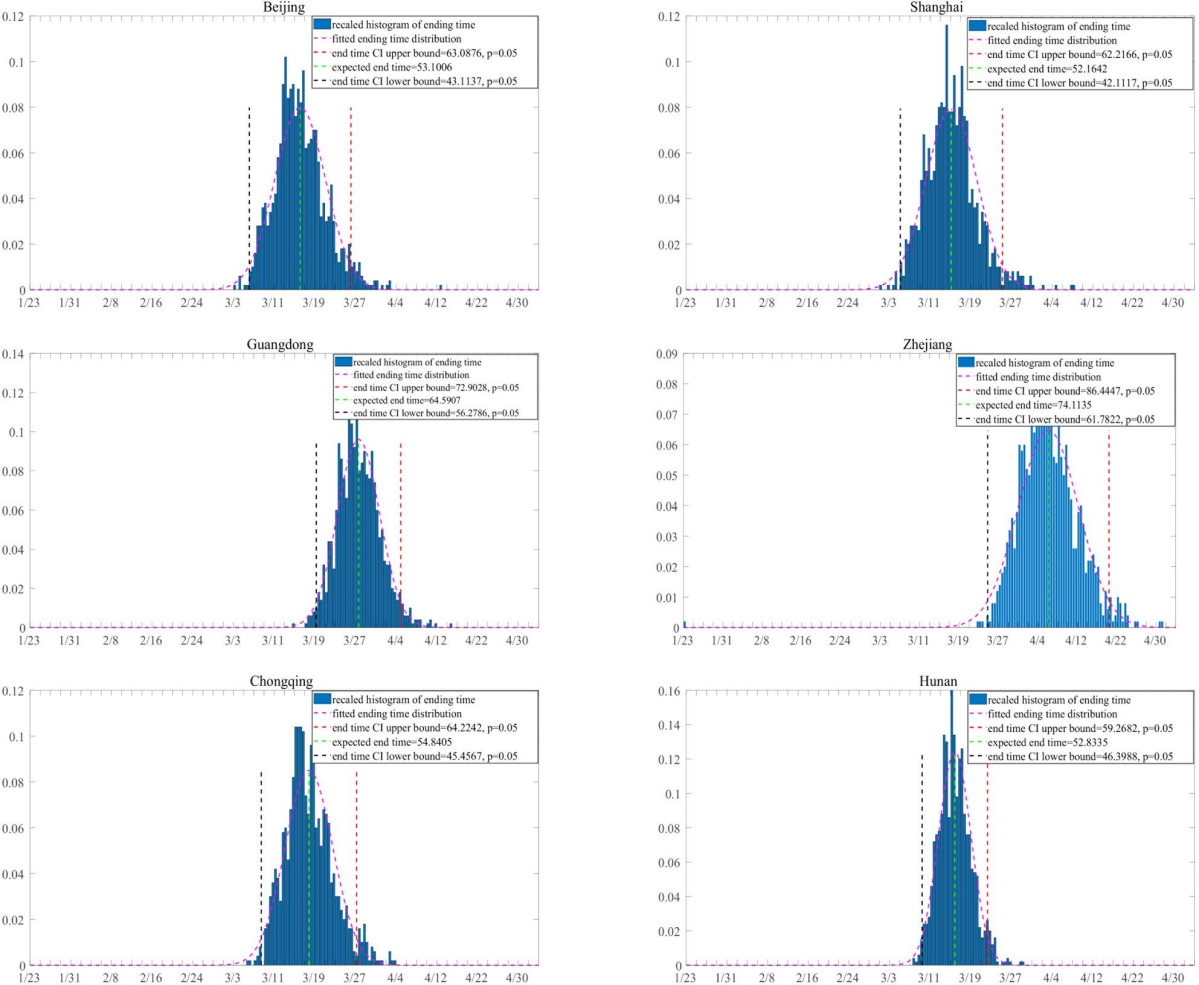
Prediction of the containment time of the outbreak with *q* = 0. The containment time of 1000 simulations is plotted as a histogram and is fitted with normal distribution for each region. The *y* axis represents the density of containment time.

Finally, we evaluate the effectiveness of the current medical tracking policy with a hypothetical controlled test by setting the probability of quarantine *q* = 0 in the proposed model with the rest of estimates unchanged. Under this setting, the epidemic would still be contained, due to the reduction of contact rate and diagnosis waiting time. However, there are significant delays in the dates of containment if *q* = 0, indicating the current medical tracking policy contribute significantly to the containment of the epidemic (see Fig. 5).

## Discussion

In this article, we propose a novel stochastic dynamic model to depict the transmission mechanism of COVID-19. In comparison with some existing dynamic models on COVID19, our model features the employment of a stochastic dynamic as well as a comprehensive account for the infectious incubation period, the asymptomatic virus carriers, and the contact tracing measure with time latency. Moreover our proposed model also sets the foundation for further studies with individual/network based models, which may not have an exact mean-field counterpart.

Based on our proposed model, we find that (1) about 30% of infections are asymptomatic which is lower than what was estimated in Tang et al.^10^ but consistent with the finding of 29.2% in Hu et al.^34^ among a small sample and 30.8% in Japanese evacuation data^35^; (2) virus carriers with symptoms are about twice as likely to pass a pathogen to others as asymptomatic virus carriers which is consistent with the finding in the study of Li et al.^36^; (3) the current containment measures are effective to reduce the contact and transmission rate; (4) the containment time of the outbreak is around late February to early March; (5) the time-varying *R*_*c*_ was estimated to be around two, which is of the same magnitude as reported in Wu et al.^11^ and Liu et al.^7^ at the beginning of the epidemic, and it drops rapidly due to the implementation of containment measures; and (6) besides the control measures on exposure rate, the current contact tracing policy contributes significantly to the containment of the epidemic. Furthermore, the proposed model fits well in other region in China, and can be easily extended to regions outside China, see Supplementary E.

With the above findings, we suggest all nations to unite in action on the agenda of containment of COVID-19 by (1) allowing testing without symptoms; (2) introducing contact tracing and quarantine; and (3) conducting measures on reducing exposure rate. Though the epidemic is under good control in China currently, we could not let our guard down. The simulated result shows that if containment measures are relaxed after 3 weeks of the containment date, the epidemic has a probability of 0.415 to resurge in Beijing, China. The probability goes up to 0.658 if containment measures are relaxed after 2 weeks of containment date and 0.878 if 1 week. The calculation of resurgence probability was inspired by Hao et al.^7^.

However, we acknowledge that there are limitations in the propose model,

1. Given the limited available data, certainly parameters, especially those “far away” from observation in the proposed model may have a potential risk of identification issues.
2. The proposed model does not apply if significant changes apply to the current epidemic control/treatment measure in the future;
3. The proposed model needs further modification if a non-negligible portion of the asymptomatic patients remain infectious after the end of quarantine.
4. Parameter estimates may lose precision if the stochastic model differs excessively from its simplification described in “Discussion” section.

In our future study, we propose to complement/generalize our current stochastic model from the following aspects:

i. *Improved medical tracking dynamic* In this ongoing work, we will introduce a more realistic dynamic for medical tracking, such that medical tracking is triggered in a more physical manner by the affirmative diagnosis of the transmission source. In such model, the quarantine process of each exposed agent depends on his/her contact history. Thus an individual based, rather than compartment dynamic is needed.
ii. *Introduction of medical service capacity* The maximal capacity of the medical service system will be considered, which might be overloaded when facing a massive outbreak. This idea was first inspired by an Amateur Demonstration.^37^ In fact, this was the key feature of what happened in Wuhan at the beginning phase of the COVID-19 outbreak. We may also allow such capacity to be time/configuration dependent to model the contribution of cabin hospitals in Wuhan.
iii. *Population flows over cities* We will model migration of people over different regions, which could have played an essential role in the spread of the epidemic before the Chinese New Year of 2020. We will also consider the transmissions on board and even allow the population flow to react on the information they have about the epidemic situation.

## Supplementary information

Supplementary Informations.

## Data Availability

Data is attached in Supplementary Table S3–S5.

## References

[1] The State Council of the People’s Republic of China. Aug 25: Daily briefing on COVID-19 cases in China (in Chinese) (accessed on 25 August 2020); http://www.gov.cn/xinwen/2020-08/25/content_5537127.htm.

[2] The State Council of the People’s Republic of China. Notice of the pneumonia outbreak prevention and control command of new coronary virus infection in Wuhan (in Chinese) (accessed on 25 August 2020); http://www.gov.cn/xinwen/2020-01/23/content_5471751.htm.

[3] Kermack WO, McKendrick AG (1927). A contribution to the mathematical theory of epidemics. Proc. R. Soc. Lond. Ser. A.

[4] Riley S (2003). Transmission dynamics of the etiological agent of SARS in Hong Kong: impact of Public Health Interventions. Science.

[5] Fraser C (2009). Pandemic potential of a strain of influenza a (H1N1): early findings. Science.

[6] Elavarasan RM, Pugazhendhi R (2020). Restructured society and environment: a review on potential technological strategies to control the COVID-19 pandemic. Sci. Total Environ..

[7] Hao X (2020). Reconstruction of the full transmission dynamics of COVID-19 in Wuhan. Nature.

[8] Kucharski AJ (2020). Early dynamics of transmission and control of COVID-19: a mathematical modelling study. Lancet Infect. Dis..

[9] Tian H (2020). An investigation of transmission control measures during the first 50 days of the COVID-19 epidemic in China. Science.

[10] Tang B (2020). Estimation of the transmission risk of the 2019-nCoV and its implication for public health interventions. J. Clin. Med..

[11] Wu JT, Leung K, Leung GM (2020). Nowcasting and forecasting the potential domestic and international spread of the 2019-nCoV outbreak originating in Wuhan, China: a modelling study. Lancet.

[12] Liu, Y., Gayle, A. A., Wilder-Smith, A. & Rocklov J. The reproductive number of COVID-19 is higher compared to SARS coronavirus. *J. Travel Med.* **27**(2) (2020). 10.1093/jtm/taaa021 PMC7074654 32052846

[13] Yang Z (2020). Modified SEIR and AI prediction of the epidemics trend of COVID-19 in China under public health interventions. J. Thoracic Disease.

[14] Kendall, D. G. Deterministic and stochastic epidemics in closed populations. In *Proceedings of the Third Berkeley Symposium on Mathematical Statistics and Probability, Volume 4: Contributions to Biology and Problems of Health*, 149–165 (University of California Press, Berkeley and Los Angeles, 1956).

[15] Athreya, K. B. & Ney, P. E. *Branching Processes* (Springer, New York), Die Grundlehren der mathematischen Wissenschaften, Band 196 (1972).

[16] Durrett, R. *Lecture Notes on Particle Systems and Percolation*. The Wadsworth & Brooks/Cole Statistics/Probability Series (Wadsworth & Brooks/Cole Advanced Books & Software, Pacific Grove, 1988).

[17] Liu, T. *et al*. Time-varying transmission dynamics of novel Coronavirus pneumonia in China. bioRxiv:2020.01.25.919787 (2020).

[18] Zhao S (2020). Preliminary estimation of the basic reproduction number of novel Coronavirus (2019-ncov) in China, from 2019 to 2020: A data-driven analysis in the early phase of the outbreak. Int. J. Infect. Dis..

[19] Chinazzi M (2020). The effect of travel restrictions on the spread of the 2019 novel Coronavirus (COVID-19) outbreak. Science.

[20] Beijing Municipal Health Commission. Situation report (in Chinese). http://wjw.beijing.gov.cn/xwzx_20031/xwfb/202003/t20200305_1679143.html.

[21] Shanghai Municipal Health Commission. Situation report (in Chinese) (accessed on 30 April 2020); http://wsjkw.sh.gov.cn/xwfb/20200222/0a10b6df11c845368af2d627d9551ed1.html.

[22] Chongqing Municipal Health Commission. Situation report (in Chinese) (accessed on 30 April 2020); http://wsjkw.cq.gov.cn/yqxxyqtb/20200221/255637.html.

[23] Health Commission of Guangdong Province. Situation report on the new coronavirus pneumonia outbreak in Guangdong province (in Chinese) (accessed on 30 April 2020); http://wsjkw.gd.gov.cn/zwyw_yqxx/content/post_2903465.html.

[24] Health Commission of Zhejiang Province. Situation report on the new coronavirus pneumonia outbreak in Zhejiang province (in Chinese) (accessed on 30 April 2020); http://www.zjwjw.gov.cn/art/2020/2/21/art_1202101_41958074.html.

[25] Health Commission of Hunan Province. Situation report on new coronavirus pneumonia outbreak in Hunan province (in Chinese) (accessed on 30 April 2020); http://wjw.hunan.gov.cn/wjw/xxgk/gzdt/zyxw_1/202002/t20200221_11187516.html.

[26] China National Bureau of Statistics. Annual data by province (in Chinese) (accessed on 30 April 2020); http://data.stats.gov.cn/easyquery.htm?cn=E0103&zb=A0301®=440000&sj=2018.

[27] National Heath Commission of the People’s Republic of China. Update on the outbreak of new Coronavirus pneumonia as of 24 hours on 12 February (in Chinese) (accessed on 30 April 2020); http://www.nhc.gov.cn/xcs/yqtb/202002/26fb16805f024382bff1de80c918368f.shtml.

[28] State Council Information Office of the People’s Republic of China. State council information office holds press conference on joint prevention and control of pneumonia outbreak with new coronavirus infection (in Chinese) (accessed on 30 April 2020); http://www.scio.gov.cn/xwfbh/xwbfbh/wqfbh/42311/42478/index.htm.

[29] Japanese Ministry of Health, Labour and Welfare. About the new-style coronavirus infectious disease which was checked in the cruise ship which is being quarantined at Yokohama port (in Japanese) (accessed on 30 April 2020); https://www.mhlw.go.jp/stf/newpage_09668.html.

[30] People’s Government of Zhejiang Province. Notice of new coronavirus pneumonia in Zhejiang province on March 3, 2020 (in Chinese) (accessed on 30 April 2020); http://www.zj.gov.cn/art/2020/3/3/art_1228996608_42060522.html.

[31] The People’s Government of Zhejiang Province. The office of the leading group for the prevention and control of new coronavirus infection in Zhejiang province issued a notice (provisional) on the prevention and control of new coronavirus infection in rural areas of Zhejiang province (in Chinese) (accessed on 30 April 2020); http://www.zj.gov.cn/art/2020/2/10/art_1228996604_41898059.html.

[32] Peng, L., Yang, W., Zhang, D., Zhuge, C. & Hong, L. Epidemic analysis of COVID-19 in China by dynamical modeling. arXiv:2002.06563. (2020).

[33] You C (2020). Estimation of the time-varying reproduction number of COVID-19 outbreak in China. Int. J. Hyg. Environ. Health.

[34] Hu Z (2020). Clinical characteristics of 24 asymptomatic infections with COVID-19 screened among close contacts in Nanjing, China. Sci. China Life Sci..

[35] Nishiura H (2020). Estimation of the asymptomatic ratio of novel coronavirus infections (COVID-19). Int. J. Infect. Dis..

[36] Li R (2020). Substantial undocumented infection facilitates the rapid dissemination of novel coronavirus (SARS-CoV2). Science.

[37] Ele Laboratory. Computer simulation programs tell you why it’s not time to go out now (in Chinese) (accessed on 30 April 2020); https://www.bilibili.com/video/av86478875/?spm_id_from=333.788.b_7265636f5f6c697374.2.

