# Supplementary material for: Prediction of the COVID-19 outbreak in China based on a new stochastic dynamic model
Source: Sci Rep. 2020 Dec 9;10:21522. doi: 10.1038/s41598-020-76630-0 (PMC7725788; doi:10.1038/s41598-020-76630-0)
Supplement: Supplementary file 1 — Supplementary Informations. [file 41598_2020_76630_MOESM1_ESM.pdf]

Prediction of the COVID-19 outbreak in China based on a new stochastic dynamic model

Yuan Zhang, Chong You, Zhenghao Cai, Jiarui Sun,
Wenjie Hu, Xiao-Hua Zhou

September 1, 2020

SUPPLEMENTARY MATERIAL

Supplementary A

The states of the proposed stochastic model are listed as follows,

- S: Susceptible.
- E: Exposed. It divided into four sub-states:
  - E_1: will become symptomatic in the future and is not traceable in medical tracking.
  - E_2: will become symptomatic in the future and is traceable in medical tracking.
  - A_1: won't become symptomatic in the future and is not traceable in medical tracking.
  - A_2: won't become symptomatic in the future and is traceable in medical tracking.
- Q: Quarantined. It divided into two sub-states:
  - E_q: will become symptomatic in the future.

- A_q: won't become symptomatic in the future.

- IN: Infected, symptomatic, but not yet admitted to hospital. They are divided into two sub-states:
  - IN_1: not traceable in medical tracking.
  - IN_2: traceable in medical tracking.

- IH: Infected, symptomatic and currently under hospitalization. divided into two sub-states:
  - IH_L: with light symptoms.
  - IH_S: with severe symptoms.

- R: Recovered. They are divided into three sub-states:
  - R_A: recover from state A_1 and A_2.
  - R_N: recover from state IN_1 and IN_2.
  - R_H: recover from state IH_L.

- D: Dead.

We denote $S(t)$, $E_1(t)$ and so on as the population sizes in the corresponding states at time t. The evolution of $\xi(t) = \{S(t), E_1(t), E_2(t), E_q(t), A_1(t), A_2(t), A_q(t), IN_1(t), IN_2(t), IH_L(t), IH_S(t), R_A(t), R_N(t), R_H(t), D(t)\}$ over time t forms a continuous time Markov Process with state space $\{0, 1, 2, ..., N\}^{15}$. The corresponding transition process can be illustrated by Figure S1.

The transition rates of the system $\xi(t)$, which uniquely determines the continuous time Markov Process in our proposed model are as follows.

- Infection:
  - $[S, E_1, ..., D] \rightarrow [S-1, E_1+1, ..., D]$ at rate $[\lambda_{IN}\theta_E(E_1+E_2) + \lambda_{IN}\theta_A(A_1+A_2) + \lambda_{IN}IN] * \frac{S}{N} * \rho * (1-q)$.
  - $[S, E_1, E_2..., R, D] \rightarrow [S-1, E_1, E_2+1..., R, D]$ at rate $[\lambda_{IN}\theta_E(E_1+E_2) + \lambda_{IN}\theta_A(A_1+A_2) + \lambda_{IN}IN] * \frac{S}{N} * \rho * q$.
  - $[S, ..., A_1, ..., R, D] \rightarrow [S-1, ..., A_1+1, ..., R, D]$ at rate $[\lambda_{IN}\theta_E(E_1+E_2) + \lambda_{IN}\theta_A(A_1+A_2) + \lambda_{IN}IN] * \frac{S}{N} * (1-\rho) * (1-q)$.

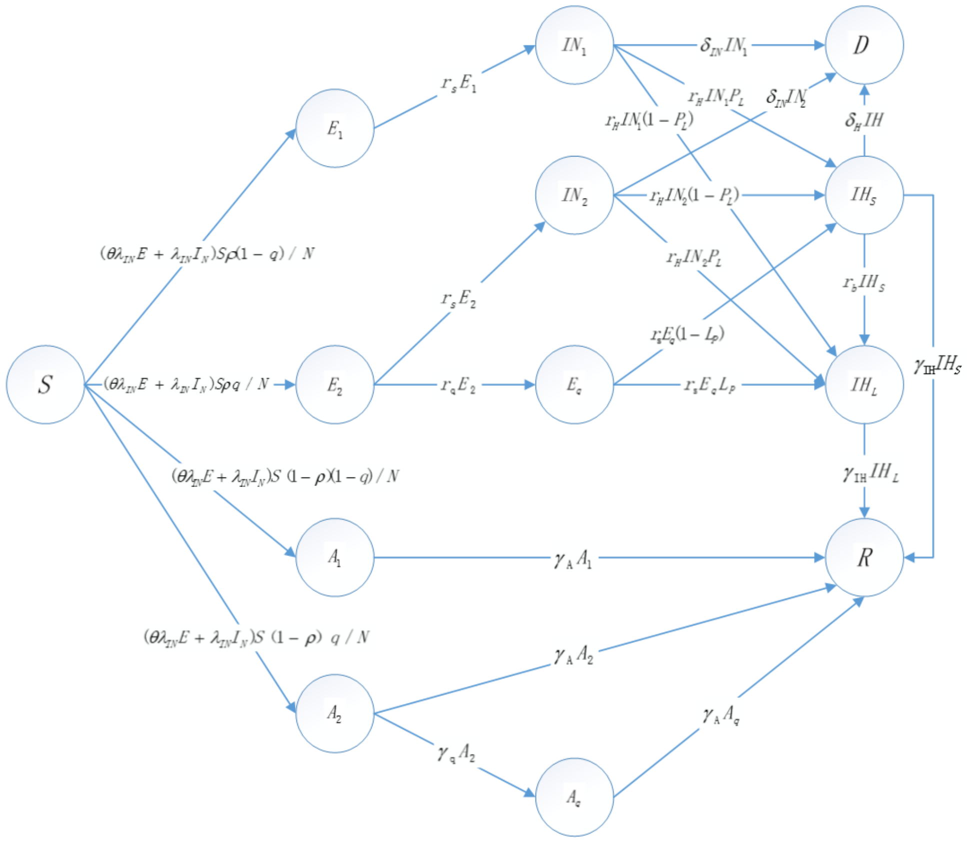

Figure S1: Process Illustration

  - $[S, ..., A_2, ..., R, D] \to [S-1, ..., A_2+1, ..., R, D]$ at rate $[\lambda_{IN}\theta_E(E_1+E_2)+\lambda_{IN}\theta_A(A_1+A_2)+\lambda_{IN}IN]*\frac{S}{N}*(1-\rho)*q$.

- Quarantine:

  - $[S, E_1, E_2, E_q..., D] \to [S, E_1, E_2-1, E_q+1..., D]$at rate $E_2 * r_q$.
  - $[S, ..., A_1, A_2, A_q..., D] \to [S, ..., A_1, A_2-1, A_q+1..., D]$at rate $A_2 * r_q$.
  - $[S, ..., IN_2, ..., IH_L, ..., D] \to [S, ..., IN_2-1, ..., IH_L+1, ..., D]$at rate $IN_2*r_q*p_1$.
  - $[S, ..., IN_2, ..., IH_S, ..., D] \to [S, ..., IN_2-1, ..., IH_S+1, ..., D]$at rate $IN_2 * r_q * (1-p_1)$.

- Symptoms onset:

  - $[S, E_1, ..., IN_1, ..., D] \to [S, E_1-1, ..., IN_1+1, ..., D]$at rate $E_1 * r_s$.
  - $[S, E_1, E_2..., IN_2, ..., D] \to [S, E_1, E_2-1, ..., IN_2+1, ..., D]$at rate $E_2 * r_s$.
  - $[S, E_1, E_2, E_q, ..., IH_L, ..., D] \to [S, E_1, E_2, E_q-1, ..., IH_L+1, ..., D]$at rate $E_q * r_s * p_1$.
  - $[S, E_1, E_2, E_q, ..., IH_S, ..., D] \to [S, E_1, E_2, E_q-1, ..., IH_S+1, ..., D]$at rate $E_q * r_s * (1-p_1)$.

- Hospitalization:

  - $[S, ..., IN_1, ..., IH_L, ..., D] \to [S, ..., IN_1-1, ..., IH_L+1, ..., D]$ at rate $IN_1 * r_H * q_1$.
  - $[S, ..., IN_1, ..., IH_S, ..., D] \to [S, ..., IN_1-1, ..., IH_S+1, ..., D]$ at rate $IN_1 * r_H * (1-q_1)$.
  - $[S, ..., IN_2, ..., IH_L, ..., D] \to [S, ..., IN_2-1, ..., IH_L+1, ..., D]$ at rate $IN_2 * r_H * q_1$.
  - $[S, ..., IN_2, ..., IH_S, ..., D] \to [S, ..., IN_2-1, ..., IH_S+1, ..., D]$ at rate $IN_2 * r_H * (1-q_1)$.

- Symptom relief:

  - $[S, ..., IH_L, IH_S, ..., D] \to [S, ..., IH_L+1, IH_S-1, ..., D]$at rate $IH_S * r_b$.

- Recovery:

- $[S, ..., A_1, ..., R_A, R_N, R_H, D] \to [S, ..., A_1 - 1, ..., R_A + 1, R_N, R_H, D]$ at rate $A_1 * \gamma_A$.
- $[S, ..., A_2, ..., R_A, R_N, R_H, D] \to [S, ..., A_2 - 1, ..., R_A + 1, R_N, R_H, D]$ at rate $A_2 * \gamma_A$.
- $[S, ..., IN_1, ..., R_A, R_N, R_H, D] \to [S, ..., IN_1 - 1, ..., R_A, R_N + 1, R_H, D]$ at rate $IN_1 * \gamma_I N$.
- $[S, ..., IN_2, ..., R_A, R_N, R_H, D] \to [S, ..., IN_2 - 1, ..., R_A, R_N + 1, R_H, D]$ at rate $IN_2 * \gamma_I N$.
- $[S, ..., IH_L, ..., R_A, R_N, R_H, D] \to [S, ..., IH_L - 1, ..., R_A, R_N, R_H + 1, D]$ at rate $IH_L * \gamma_I H$.

- Death:
  - $[S, ..., IN_1, ..., D] \to [S, ..., IN_1 - 1, .., D + 1]$at rate $IN_1 * \delta_{IN}$.
  - $[S, ..., IN_2, ..., D] \to [S, ..., IN_2 - 1, .., D + 1]$at rate $IN_2 * \delta_{IN}$.
  - $[S, ..., IH_S, ..., D] \to [S, ..., IH_S - 1, .., D + 1]$at rate $IH_S * \delta_{IH}$.

From a functional analysis point of view, the stochastic dynamic model defined above can be equivalently described with its Markov Semigroup or infinitesimal generator. See [11, 5] for details. From those operators, it is natural for us to consider the following **Mean-field Differential Equation System** that serves as a deterministic counterpart of the stochastic model,

$$
\begin{aligned}
&\tilde{S}(t)' = -\frac{\tilde{S}(t)}{N}\left(\lambda_{IN}\theta_E\tilde{E}(t) + \lambda_{IN}\theta_A\tilde{A}(t) + \lambda_{IN}\tilde{IN}(t)\right),\\
&\tilde{E}_1(t)' = \frac{\tilde{S}(t)}{N}\left(\lambda_{IN}\theta_E\tilde{E}(t) + \lambda_{IN}\theta_A\tilde{A}(t) + \lambda_{IN}\tilde{IN}(t)\right)\rho\,(1-q) - r_s\tilde{E}_1(t),\\
&\tilde{E}_2(t)' = \frac{\tilde{S}(t)}{N}\left(\lambda_{IN}\theta_E\tilde{E}(t) + \lambda_{IN}\theta_A\tilde{A}(t) + \lambda_{IN}\tilde{IN}(t)\right)\rho q - (r_s + r_q)\tilde{E}_2(t),\\
&\tilde{E}_q(t)' = r_q\tilde{E}_2(t) - r_s\tilde{E}_q(t),\\
&\tilde{A}_1(t)' = \frac{\tilde{S}(t)}{N}\left(\lambda_{IN}\theta_E\tilde{E}(t) + \lambda_{IN}\theta_A\tilde{A}(t) + \lambda_{IN}\tilde{IN}(t)\right)(1-\rho)\,(1-q) - \gamma_A\tilde{A}_1(t),\\
&\tilde{A}_2(t)' = \frac{\tilde{S}(t)}{N}\left(\lambda_{IN}\theta_E\tilde{E}(t) + \lambda_{IN}\theta_A\tilde{A}(t) + \lambda_{IN}\tilde{IN}(t)\right)(1-\rho)\,q - (\gamma_A + r_q)\tilde{A}_2(t),\\
&\tilde{A}_q(t)' = r_q\tilde{A}_2(t) - \gamma_A\tilde{A}_q(t),\\
&\tilde{IN}_1(t)' = r_s\tilde{E}_1(t) - (r_H + \gamma_{IN} + \delta_{IN})\,\tilde{IN}_1(t),\\
&\tilde{IN}_2(t)' = r_s\tilde{E}_2(t) - (r_H + r_q + \gamma_{IN} + \delta_{IN})\,\tilde{IN}_2(t),\\
&\tilde{IH}_L(t)' = p_1\left[r_H\tilde{IN}_1(t) + (r_q + r_H)\,\tilde{IN}_2(t) + r_s\tilde{E}_q(t)\right] + r_b\tilde{IH}_S(t) - \gamma_H\tilde{IH}_L(t),\\
&\tilde{IH}_S(t)' = (1-p_1)\left[r_H\tilde{IN}_1(t) + (r_q + r_H)\,\tilde{IN}_2(t) + r_s\tilde{E}_q(t)\right] - r_b\tilde{IH}_S(t) - \delta_H\tilde{IH}_S(t),\\
&\tilde{R}_A(t)' = \gamma_A\tilde{A}(t),\\
&\tilde{R}_N(t)' = \gamma_{IN}\tilde{IN}(t),\\
&\tilde{R}_H(t)' = \gamma_H\tilde{IH}_L(t),\\
&\tilde{D}(t)' = \delta_{IN}\tilde{IN}(t) + \delta_H\tilde{IH}_S(t),
\end{aligned}
$$

where $\tilde{S}(t)$ is the deterministic counterpart of $S(t)$ and the same notation applies to the rest. Intuitively, the mean-field ODE system above serves as a degenerated case of our stochastic model, where all randomness has been averaged out. When the differential equations are linear, which is not true in our case, the ODE also describes the evolution of the expectation of the stochastic system (Dynkin's Formula, see [4] Section 4.4 for example). Besides, according to [9] and [10], we may see that this deterministic model can also serve, under a much weaker condition, as a scaling approximation of the stochastic one after recaled by the renormalizing factor N. To be more specific, the renormalized random path $\xi_N(t)/N$ as a stochastic process is "almost" deterministic, and fluctuates closely around the deterministic trajectory $\tilde{\xi}_N(t)/N$ if the total population N is large and the renormalized initial

values $\xi_N(0)/N$ and $\tilde{\xi}_N(0)/N$ are close to each other, where $\tilde{\xi}(t)$ denotes the determinsitic counterpart of $\xi(t)$, and $\tilde{\xi}_N(t)$ and $\xi_N(t)$ be copies of the deterministic and stochastic models with total population N. Using mathematical language, with probability one there is pathwise convergence

$$\lim_{N\to\infty} \max_{t\leq t_0} \frac{\|\xi_N(t) - \tilde{\xi}_N(t)\|}{N} = 0 \tag{1}$$

for all $t_0 \in [0, \infty)$. However, we would like to specifically bring to reader's attention that, NO MATTER the size of the total population N, as long as the size of epidemic outbreak is NOT comparable to N, the convergence above in (1) DOES NOT imply that the un-renormalized $\xi(t)$ is non-random by itself or the epidemic involved populations. For example, $E_1(t)$ and $\tilde{E}_1(t)$ are close to each other in any sense without the rescaling factor. Actually the fact that the size of epidemic outbreak is not comparable to N itself already implies that

$$\lim_{N\to\infty} \frac{\xi_N(t)}{N} = \lim_{N\to\infty} \frac{\tilde{\xi}_N(t)}{N} = (1, 0, 0, \cdots, 0), \tag{2}$$

which perfectly satisfies (1) while at the same time provides no information on whether the actual values of $E_1(t)$ and $\tilde{E}_1(t)$ are close to each other. To summarize, when the size of epidemic outbreak is NOT comparable to N, the stochastic model possesses intrinsic randomness, and may not be well approximated by a deterministic ODE in any non-degenerate sense.

Supplementary B

In the state-collapsed version of the stochastic process (see Figure 1), the D is removed as fatality rate is extremely low in all the selected regions and the collected data could not provide reliable estimations of the death rates, and only recoveries from hospital, R_H, is considered. Furthermore, states E_1, E_2, A_1 and A_2 are collapsed into E, states IN_1 and IN_2 are collapsed into IN, and IH_L and IH_S are collapsed into IH, which would ease the identifications of the initial values in the model.

Supplementary C

Table S1: Priors for parameter estimation and values for prefixed parameters

Priors for estimation		
Parameter	Prior	Notes
ρ	Uniform(0,1)	ρ is the same for all provinces/cities.
θ_E	Uniform(0,1)	θ is the same for all provinces/cities.
λ_{IN}	Uniform(0,0.7)	Combining with the prefixed parameter r_H, the upper bound of λ_{IN} here means R_0 is at least 0.7*5, about 3.5,which is a large enough upper bound based on existing estimates for R_0. [16]
q	Uniform(0,1)	
γ_{IH}	Uniform(1/30,1)	It is assumed patients with light symptoms should recover in a month in hospital, which means γ_{IH} is larger than 1/30.
a	Uniform(0,0.2)	Existing data show new infections have begun to decrease. This means the speed of infection has droped to a relatively low value, so a should be quite small.
b	Uniform(0,1)	
Prefixed parameters		
Parameter	value	notes
r_H	1/5.6	r_H,r_s,r_q can be prefixed through the average time from symptoms onset to diagnosis, the mean incubation period and mean difference between infectious period and serial interval shown in [17]. We assume that r_q increase to 1/3.6 (which means the expected difference between infectious period and serial decreases by 3 days) due to improvement in testing means and control measures after these measures are taken.
r_s	1/5.3	
r_q	t<7: 1/6.6	
	t≥7:1/3.6	

Table S2: Initial Values

Initial values			
City	Parameter	Prior	Notes
Beijing	$E(0)$	Uniform(0,179)	Upper bound for IN(0) is obtianed from the new confirmed cases in the first 5 days. Upper bound for E(0) is obtained from a coarse estimate based on a conservative R_0 shown in [16].
	$IN(0)$	Uniform(0,66)	
Shanghai	$E(0)$	Uniform(0,136)	
	$IN(0)$	Uniform(0,50)	
Guangdong	$E(0)$	Uniform(0,424)	
	$IN(0)$	Uniform(0,156)	
Zhejiang	$E(0)$	Uniform(0,443)	
	$IN(0)$	Uniform(0,163)	
Chongqing	$E(0)$	Uniform(0,275)	
	$IN(0)$	Uniform(0,101)	
Hunan	$E(0)$	Uniform(0,364)	
	$IN(0)$	Uniform(0,134)	

Supplementary D

In this study, the evolution of R_c is approximated by the ratio between in-and-out flows of the active virus carriers in a given time period, that is, for a time interval of length Δt, say $[t, t + \Delta t]$, we keep tracking the transitions that lead to the increase/decrease of the active virus carrier population E and IN, with their accumulative numbers recorded.

Supplementary E

Supplementary F

date	Beijing	Shanghai	Chongqing	Guangdong	Zhejiang	Hunan
2020.01.22	14	16	9	32	10	9
2020.01.23	26	20	27	53	43	24
2020.01.24	36	33	57	78	62	43
2020.01.25	51	40	75	98	104	69
2020.01.26	68	53	110	146	128	100
2020.01.27	80	66	110	188	173	143
2020.01.28	91	80	147	241	296	221
2020.01.29	111	101	165	311	428	277
2020.01.30	132	128	206	393	537	332
2020.01.31	156	153	238	520	599	389
2020.02.01	183	169	262	604	661	463
2020.02.02	212	193	300	683	724	521
2020.02.03	228	208	337	797	829	593
2020.02.04	253	233	366	870	895	661
2020.02.05	274	254	389	944	954	711
2020.02.06	297	269	411	1018	1006	772
2020.02.07	315	281	426	977	1048	803
2020.02.08	327	292	446	1120	1075	838
2020.02.09	337	295	468	1151	1092	879
2020.02.10	342	302	484	1177	1117	912
2020.02.11	352	306	505	1219	1131	946
2020.02.12	366	313	518	1241	1145	968
2020.02.13	372	318	529	1261	1155	988
2020.02.14	375	326	537	1294	1162	1001
2020.02.15	380	328	544	1316	1167	1004
2020.02.16	381	331	551	1322	1171	1006
2020.02.17	387	333	553	1328	1172	1007
2020.02.18	393	333	555	1331	1173	1008
2020.02.19	395	333	560	1332	1175	1010
2020.02.20	396	334	567	1333	1203	1011
2020.02.21	399	334	572	1339	1205	1013

Table S3: Culmulative confirmed cases of COVID-19. Data for the six regions is available at [1, 3, 2, 12, 15, 13].

Date	Beijing	Shanghai	Chongqing	Guangdong	Zhejiang	Hunan
2020.01.22	0	0	0	0	1	0
2020.01.23	0	0	0	2	1	0
2020.01.24	1	1	0	2	1	0
2020.01.25	2	1	0	2	1	0
2020.01.26	2	1	0	2	1	0
2020.01.27	2	3	0	4	3	0
2020.01.28	2	4	0	5	3	0
2020.01.29	4	5	0	6	4	0
2020.01.30	5	5	1	11	9	2
2020.01.31	5	9	1	12	15	3
2020.02.01	5	10	3	12	23	8
2020.02.02	9	10	7	14	36	16
2020.02.03	12	10	9	20	48	22
2020.02.04	24	15	14	32	63	35
2020.02.05	31	15	15	49	81	56
2020.02.06	33	25	24	52	98	91
2020.02.07	34	30	31	97	127	119
2020.02.08	37	41	39	125	173	159
2020.02.09	37	44	51	143	201	186
2020.02.10	48	48	66	181	250	213
2020.02.11	56	53	79	241	279	263
2020.02.12	68	57	102	284	327	312
2020.02.13	79	62	128	332	367	352
2020.02.14	97	90	152	386	409	389
2020.02.15	105	124	184	436	437	430
2020.02.16	114	140	207	473	470	467
2020.02.17	122	161	225	530	514	501
2020.02.18	145	177	254	571	544	542
2020.02.19	153	186	274	619	609	578
2020.02.20	169	199	299	664	647	638
2020.02.21	178	221	316	720	694	670

Table S4: Cumulative recoveries of COVID-19. The data for the six regions are available at [1, 3, 2, 12, 15, 13].

City/Province	Permanent population
Beijing	21,540,000
Shanghai	24,240,000
Guangdong	113,460,000
Chongqing	31,020,000
Hunan	68,990,000
Zhejiang	57,370,000

Table S5: Population of permanent residents . The data for the six regions are available at [14] .

References

[1] Beijing Municipal Health Commission. Situation Report (in Chinese). Available at http://wjw.beijing.gov.cn/xwzx_20031/xwfb/202003/t20200305_1679143.html, 2020.

[2] Chongqing Municipal Health Commission. Situation Report (in Chinese). Available at http://wsjkw.cq.gov.cn/yqxxyqtb/20200221/255637.html, 2020.

[3] Shanghai Municipal Health Commission. Situation Report (in Chinese). Available at http://wsjkw.sh.gov.cn/xwfb/20200222/0a10b6df11c845368af2d627d9551ed1.html, 2020.

[4] Giuseppe Da Prato and Jerzy Zabczyk. Stochastic Equations in Infinite Dimensions, volume 152 of Encyclopedia of Mathematics and its Applications. Cambridge University Press, Cambridge, second edition, 2014.

[5] Stewart N. Ethier and Thomas G. Kurtz. Markov Processes. Wiley Series in Probability and Mathematical Statistics: Probability and Mathematical Statistics. John Wiley & Sons, Inc., New York, 1986. Characterization and convergence.

[6] KPMG International. Germany – measures to Combat COVID-19 Become More Intense. Available at https://home.kpmg/xx/en/home/insights/2020/03/flash-alert-2020-106.html, 2020.

[7] Aaron A. King, Edward L. Ionides, Carles Martinez Bretó, Stephen P. Ellner, Matthew J. Ferrari, Bruce E. Kendall, Michael Lavine, Dao Nguyen, Daniel C.

Reuman, Helen Wearing, and Simon N. Wood. pomp: Statistical Inference for Partially Observed Markov Processes, 2020. R package, version 2.7.

[8] Aaron A. King, Dao Nguyen, and Edward L. Ionides. Statistical Inference for Partially Observed Markov Processes via the R package pomp. Journal of Statistical Software, 69(12):1–43, 2016.

[9] Thomas G Kurtz. Solutions of Ordinary Differential Equations as Limits of Pure Jump Markov Processes. Journal of Applied Probability, 7(1):49–58, 1970.

[10] Thomas G Kurtz. Limit Theorems for Sequences of Jump Markov Processes Approximating Ordinary Differential Processes. Journal of Applied Probability, 8(2):344–356, 1971.

[11] Thomas M. Liggett. Interacting Particle Systems, volume 276 of Grundlehren der Mathematischen Wissenschaften. Springer-Verlag, New York, 1985.

[12] Health Commission of Guangdong Province. Situation Report on New Coronavirus Pneumonia Outbreak in Guangdong province (in Chinese). Available at http://wsjkw.gd.gov.cn/zwyw_yqxx/content/post_2903465.html, 2020.

[13] Health Commission of Hunan Province. Situation Report on new coronavirus pneumonia outbreak in hunan province (in Chinese). Available at http://wjw.hunan.gov.cn/wjw/xxgk/gzdt/zyxw_1/202002/t20200221_11187516.html, 2020.

[14] China National Bureau of Statistics. Annual Data by Provinces (in Chinese). Available at http://data.stats.gov.cn/easyquery.htm?cn=E0103&zb=A0301&reg=440000&sj=2018, 2018.

[15] Health Commission of Zhejiang Province. Situation Report on new coronavirus pneumonia outbreak in Guangdong province (in Chinese). Available at http://www.zjwjw.gov.cn/art/2020/2/21/art_1202101_41958074.html, 2020.

[16] Joseph T Wu, Kathy Leung, and Gabriel M Leung. Nowcasting and Forecasting the Potential Domestic and International Spread of the 2019-nCoV Outbreak Originating in Wuhan, China: a modelling study. Lancet, 2020.

[17] Chong You, Yuhao Deng, Wenjie Hu, Jiarui Sun, Qiushi Lin, Feng Zhou, Cheng Heng Pang, Yuan Zhang, Zhengchao Chen, and Xiao-Hua Zhou. Estimation of the time-varying reproduction number of COVID-19 outbreak in China. International Journal of Hygiene and Environmental Health, 228:113555, 2020.
